# Effects of multi-phase nutritional programs associated with balanced digestible amino acid profiles on the productive performance of Japanese and European Quails

**DOI:** 10.1016/j.psj.2026.107706

**Published:** 2026-08-26

**Authors:** Adiel Vieira de Lima, Danilo Vargas Gonçalves Vieira, Ricardo Romão Guerra, Raiane dos Santos Silva, Carlos Henrique do Nascimento, Paloma Eduarda Lopes de Souza, Aline Beatriz Rodrigues, Raul da Cunha Lima Neto, Matheus Ramalho de Lima, Apolônio Gomes Ribeiro, Humberto de Araújo Brito Filho, Vânia Maria Bernardo de Araújo, José de Arimatéia de Freitas Pinto, Lucas Rannier Ribeiro Antonino Carvalho, Fernando Guilherme Perazzo Costa

**Affiliations:** a Department of Animal Science, Federal University of Paraíba, Areia, Paraíba, Brazil; b Federal University of Northern Tocantins, Department of Animal Science, Araguaína, Tocantins, Brazil; c Department of Veterinary Sciences, Federal University of Paraíba, Areia, Paraíba, Brazil; d Federal University of Western Pará, Institute of Biodiversity and Forests, Santarém, PA, Brazil; e Federal Rural University of the Semi-Arid, Department of Animal Science, Mossoró, RN, Brazil; f Department of Physiology and Pharmacology, Karolinska Institutet, Biomedicum 5B, Solnavägen 9, S-171 77, Stockholm, Sweden

**Keywords:** Digestible amino acids, Feed efficiency, Carcass yield, Egg quality, Nutrient utilization

## Abstract

Nutritional requirements of Japanese and European quails vary with genetic background, production phase, and production purpose, emphasizing the need to match nutrient supply with physiological demands. Multi-phase programs with balanced digestible amino acid profiles may improve nutrient utilization and productive efficiency. This study evaluated different nutritional programs for Japanese and European quails based on the recommendations of NRC (1994), Silva and Costa (2009), Rostagno et al. (2011, 2017, 2024), and commercial guidelines. A total of 1,800 quails were distributed into three experiments involving growing Japanese quails, laying Japanese quails, and growing European meat-type quails. In each experiment, six nutritional programs were evaluated, with 10 replicates of 10 birds per treatment. Performance, egg quality, feed efficiency, and carcass traits were evaluated according to the experimental phase and type of quail. Data were subjected to analysis of variance, and means were compared using Tukey’s test at a 5% probability level (P < 0.05). In growing Japanese quails, the Rostagno et al. (2024) program resulted in the lowest feed intake (12.90 g/quail/day) and feed conversion ratio (3.37 g/g), representing reductions of 15.0% and 6.9%, respectively, compared with the highest values among treatments. In laying Japanese quails, the Rostagno et al. (2024) program resulted in the highest egg production (89.88%) and egg mass (9.51 g/quail/day), as well as the highest specific gravity (1.070 g/cm³) and eggshell percentage (8.90%). In European quails, the multi-phase program based on Rostagno et al. (2024) resulted in lower feed intake (17.3 g/quail/day), better feed conversion (2.6 g/g), and greater carcass yield (75.2%) than the NRC (1994) program. Overall, the Rostagno et al. (2024) program provided the most consistent improvements in feed efficiency across growing quails and enhanced productive performance and selected egg quality traits in laying Japanese quails. These findings highlight the importance of updating nutritional programs according to physiological stage, genetic potential, and productive purpose.

## Introduction

The nutritional requirements of Japanese and European quail have been continuously updated in recent years due to genetic advances in strains, environmental rearing conditions, and the pursuit of greater productive efficiency.

Recent studies have demonstrated that adjustments in levels of protein, metabolizable energy, digestible amino acids, and minerals directly influence performance, egg quality, carcass yield, and the physiological responses of birds (Ashour et al., 2024; Lima et al., 2024; Vieira et al., 2025; Adhikari et al., 2025). In addition, recent research highlights that more precise nutritional strategies, associated with the use of digestible amino acids, factorial models, and multistage feeding programs, contribute to improved feed efficiency, better nutrient deposition, and reduced production costs in Japanese and European quail (Ghiasvand et al., 2025; Souza et al., 2025; Vieira et al., 2026).

In European quail intended for meat production, studies emphasize the importance of nutritional balancing. Lima et al. (2024) observed that nutritional plans adjusted for energy and protein improved productive performance and feed efficiency in quail subjected to high environmental temperatures. Furthermore, Correa et al. (2025) highlighted that nutritional strategies involving fiber and functional additives may influence hematological and metabolic parameters in European quail.

Vieira et al. (2025) updated the estimates of calcium and available phosphorus requirements for growing Japanese quail, demonstrating differences in mineral retention according to bird age. Ashour et al. (2024) reported improved productive responses in birds fed approximately 21% crude protein and 2,900 kcal/kg during the laying phase and stated that nutritional requirements may vary according to the season, with higher energy demands observed during periods of elevated temperatures.

Digestible amino acids have also received increasing attention in quail nutrition. Souza et al. (2025) reported that DL-methionine supplementation contributed to improvements in performance and egg quality in quail subjected to heat stress. In another study, Ghiasvand et al. (2025) evaluated digestible arginine levels for meat-type quail and observed positive effects on growth and immune response. Similarly, Ndunguru et al. (2025) found that supplementation of essential amino acids, particularly methionine and leucine, enhanced growth and protein metabolism in birds.

In addition to experimental studies, recent reviews have consolidated important information on quail nutritional requirements under tropical conditions. The review proposed by Altine et al. (2016) compiled updated data on protein, energy, amino acids, and minerals for different productive phases. Likewise, Adhikari et al. (2025) highlighted advances in protein and amino acid research in poultry, emphasizing the importance of the ideal protein concept to maximize performance and sustainability.

Recently, the use of nonlinear models and the factorial method has gained prominence in determining nutritional requirements and developing feeding programs for Japanese and European quail. These models allow for a more accurate description of body growth curves, as well as protein, fat, and mineral deposition across different production phases, enabling more precise estimates of nutritional requirements according to the physiological demands of birds. Studies using mathematical models such as Gompertz, Logistic, Richards, Brody, and von Bertalanffy have demonstrated a high capacity to predict growth and body composition in quail (Marcato et al., 2008; Raji et al., 2014; Mota et al., 2015; Grieser et al., 2015; Grieser et al., 2018; Ribeiro et al., 2020; Başar and Balcıoğlu, 2023; Vieira et al., 2026).

In Japanese quail, Vieira et al., 2020a, Vieira et al., 2020b quantified energy and protein requirements during the starter and growing phases using the comparative slaughter methodology associated with factorial modeling, observing differences in maintenance and growth requirements between production phases, as well as the influence of environmental temperature on bird metabolism. Subsequently, Bourdon et al. (2023a, b) developed prediction models for crude protein and metabolizable energy in laying Japanese quail, considering maintenance, body weight gain, and egg mass production, demonstrating that nutritional requirements vary according to metabolic body weight and productive potential of birds.

More recently, Vieira et al. (2026) demonstrated that nonlinear models such as Gompertz, Logistic, and Richards present a high capacity to predict body growth and the deposition of protein, fat, and minerals in Japanese quail. The authors verified that peaks of protein and lipid deposition occur at different stages of development, indicating that multistage feeding programs may improve nutrient utilization, feed efficiency, and productive longevity of birds.

In European meat-type quail (*Coturnix coturnix*), Rossi et al. (2017) found that the Gompertz model provided the best fit to describe body growth of different strains, highlighting differences between sexes and genetic groups regarding asymptotic weight and maturity rate. Ribeiro et al. (2020) compared different nonlinear models in European quail subjected to distinct levels of metabolizable energy and crude protein and observed that the Richards and Gompertz models were the most suitable to predict bird growth. According to the authors, differences in growth curves among strains and feeding programs reinforce the need to adopt specific nutritional plans for each production phase and genetic potential.

Marcato et al. (2008) and Mota et al. (2015) emphasized that the prediction of body deposition through mathematical models may assist in the formulation of more precise diets, contributing to improved feed efficiency and reduced production costs. Thus, nonlinear models have been used as important tools to understand the dynamics of growth and nutrient deposition in quail, providing a more accurate basis for the development of modern nutritional plans adjusted to different production phases.

Overall, studies indicate a growing trend toward more precise nutritional formulations for Japanese and European quail, considering factors such as thermal environment, age, production purpose, body nutrient deposition, and the use of digestible amino acids. These advances contribute to improved feed efficiency, cost reduction, and better productive performance of birds.

Considering the advances in quail nutrition, it is observed that nutritional requirements vary according to strain, production purpose, bird age, and environmental conditions, especially under heat stress situations. Currently, diet formulation for quail is mainly based on consolidated references such as Silva and Costa (2009), Rostagno et al. (2011, 2017, 2024), NRC (1994), and commercial strain manuals, which present methodological differences in estimating nutritional requirements. These variations reinforce the importance of practical validation of nutritional recommendations for different production systems and rearing conditions. Furthermore, the use of nonlinear models applied to growth and nutrient deposition allows a more precise understanding of bird requirements throughout different production phases, contributing to the development of more specific and efficient nutritional plans.

Thus, the evaluation of different nutritional plans becomes essential to identify feeding strategies better suited to current production conditions, contributing to improvements in zootechnical performance, feed efficiency, product quality, and economic viability of modern quail production.

## Materials and methods

### Animal husbandry and management

This study was conducted at the commercial facilities of Holanda Avicultura, located in the municipality of Macaíba, Rio Grande do Norte, Brazil. All protocols and procedures followed animal welfare guidelines and were approved by the Ethics Committee on the Use of Animals (CEUA) of the Federal Rural University of the Semi-Arid (UFERSA) under protocol no. 25/2025.

A total of 1,800 quails straight-run birds for the growth and meat-type experiments, and exclusively females for the egg-production experiment was used in the study. Birds were housed in a masonry shed with natural ventilation and side curtains. These birds were obtained at one day of age and managed according to standard husbandry recommendations. The Birds were housed in galvanized wire cages measuring 100 × 45 × 45 cm (stocking density of 22.2 birds/m²), and during the egg-production phase, quails were allocated in galvanized wire cages measuring 33 × 34 × 20 cm (stocking density of 89.1 birds/m²). All cages were equipped with trough-type feeders and nipple drinkers with *ad libitum* access to clean water.

Throughout the experimental period, the birds remained in good health, with no clinical signs of disease observed. Mortality was monitored daily, and no mortality was recorded in any of the experiments.

The lighting program adopted in the growth experiments followed the recommendations of the Fujikura Genetics Manual: 24 hours of continuous light from day 1 to 21 of age to ensure initial feed intake, followed by a gradual reduction to 13 hours of light daily between day 21 and 36 to prevent premature sexual maturity. For the egg-production experiment (adult birds at 15 weeks of age), a constant lighting program of 16 hours of light and 8 hours of dark (16L:8D) was provided throughout the 105-day experimental period, using supplemental artificial light over the 12-hour natural photoperiod.

### Experimental diets and design

The experiment was conducted to evaluate the overall efficacy of different integrated nutritional programs plans proposed by Silva and Costa (2009), Rostagno et al. (2011, 2017, 2024), NRC (1994), and commercial manuals, tested in their entirety as complete technological packages. All experimental diets were provided in mash form and formulated based on the respective reference nutritional matrices. Samples of the main ingredients used in the feeds (corn, soybean meal, and wheat bran) were previously subjected to near-infrared spectroscopy (NIRS) analysis to verify their nutritional profile and adjust formulations.

The allocation of cages across the batteries followed a systematic pattern of position blocks arranged in an alternating serpentine layout (e.g., T1R1, T2R1... T6R1; T6R2... T1R2), ensuring uniform distribution of treatments throughout the facility and neutralizing potential microenvironmental effects, such as light gradients, cage height, and air currents. Each cage containing 10 birds was explicitly defined as the experimental unit for all performance, feed intake, and feed conversion ratio analyses. The selection of 10 replicates with 10 birds per cage (100 birds per treatment and 600 birds per experiment) was based on the standard sampling framework for quail research, ensuring low experimental error and high statistical power for detecting significant differences.

Experiment A (Table 1 and Fig. 1.) – Japanese quails (rearing and growing phases): A total of 600 one-day-old Japanese quails straight-run, with an average initial body weight of 7.46 ± 0.11 g was used, distributed into six treatments with 10 replicates of 10 birds each (100 birds per treatment). The treatments were: T1 – NRC (1994), from 1 to 42 d; T2 – Silva and Costa (2009), from 1 to 21 and 22 to 42 d; T3 – Rostagno et al. (2011), from 1 to 42 d; T4 – Rostagno et al. (2017), from 1 to 14 and 15 to 35 d; T5 – Rostagno et al. (2024), from 1 to 14 and 15 to 35 d; and T6 – Commercial Manual, from 1 to 20 and 21 to 38 d of age. The duration of each program strictly followed the phase periods and closing ages recommended in their respective original literature and manuals, reflecting the genetic progress captured in more recent tables.

**Table 1:** Nutritional requirements of growing Japanese quails.

Ingredients	Composition of diets (g/kg)									
	T1	T2		T3	T4		T5		T6	
Corn (7.88%)	535.1	501.1	564.5	590.9	503.5	558.2	488.2	519.3	511.3	561.1
Soybean meal	426.2	453.5	378.3	373.9	439.3	401.1	470.7	454.7	419.5	385.3
Soybean oil	8.9	14.5	27.8	1.9	17.5	7.0	9.1	1.5	31.3	15.8
Limestone	10.7	11.2	9.5	10.9	10.9	9.2	4.5	4.9	8.6	9.5
Dicalcium phosphate	9.6	10.5	10.1	14.1	21.1	16.8	16.6	9.0	21.1	20.3
Salt	3.43	5.69	5.71	4.10	4.82	5.05	4.80	5.03	4.70	4.45
L-Lysine	0.91	—	—	0.36	—	—	0.84	2.21	—	—
DL-Methionine	1.01	1.30	1.39	1.57	0.89	0.67	2.67	0.94	1.47	1.52
L-Threonine	1.98	0.13	0.63	0.32	—	—	0.58	0.36	—	—
Mineral premix	1.0	1.0	1.0	1.0	1.0	1.0	1.0	1.0	1.0	1.0
Vitamin premix	1.0	1.0	1.0	1.0	1.0	1.0	1.0	1.0	1.0	1.0
Total	1000	1000	1000	1000	1000	1000	1000	1000	1000	1000
Chemical composition (g/kg)										
Nutrients	T1*	T2		T3	T4		T5		T6	
Crude protein	240.00	250.00	220.00	220.00	240.36	230.01	250.70	250.20	230.50	220.30
Metab. energy (MJ/kg)	12.13	12.13	12.76	12.13	12.13	12.13	11.92	11.92	12.44	12.34
Crude fiber	26.2	26.7	24.87	25.0	26.1	25.6	27.1	27.0	25.5	25.0
Calcium	8.00	8.50	7.50	9.00	10.92	9.11	7.52	5.74	10.00	10.00
Available phosphorus	3.00	3.20	3.00	3.75	5.13	4.28	4.35	2.93	5.10	4.90
Sodium	1.50	2.40	2.40	1.76	2.05	2.14	2.05	2.14	2.00	1.90
Chloride	2.74	4.07	4.10	3.16	3.54	3.71	3.54	3.69	3.47	3.35
Potassium	9.51	9.90	8.73	8.73	9.65	9.13	10.18	9.98	9.31	8.85
Digestible lysine	13.00	11.90	10.91	11.20	12.38	11.49	14.00	11.50	11.40	11.08
Digestible methionine	4.24	4.62	4.38	4.57	4.14	3.78	6.06	4.30	4.61	4.54
Digestible M + C	7.50	8.00	7.40	7.60	7.44	6.93	9.52	7.71	7.80	7.60
Digestible threonine	10.20	8.70	8.20	7.90	8.36	7.92	9.38	9.00	7.40	7.68

T1 - NRC (1994) – 1 to 42 days; T2 - Silva and Costa (2009) – 1 to 21 and 22 to 42 days; T3 - Rostagno et al. (2011) – 1 to 42 days; T4 - Rostagno et al. (2017) – 1 to 14 and 15 to 35 days; T5 - Rostagno et al. (2024) – 1 to 14 and 15 to 35 days; T6 - Commercial strain manual – 1 to 20 and 21 to 38 days of age. *According to NRC recommendations expressed as total amino acids.

**Fig. 1:**
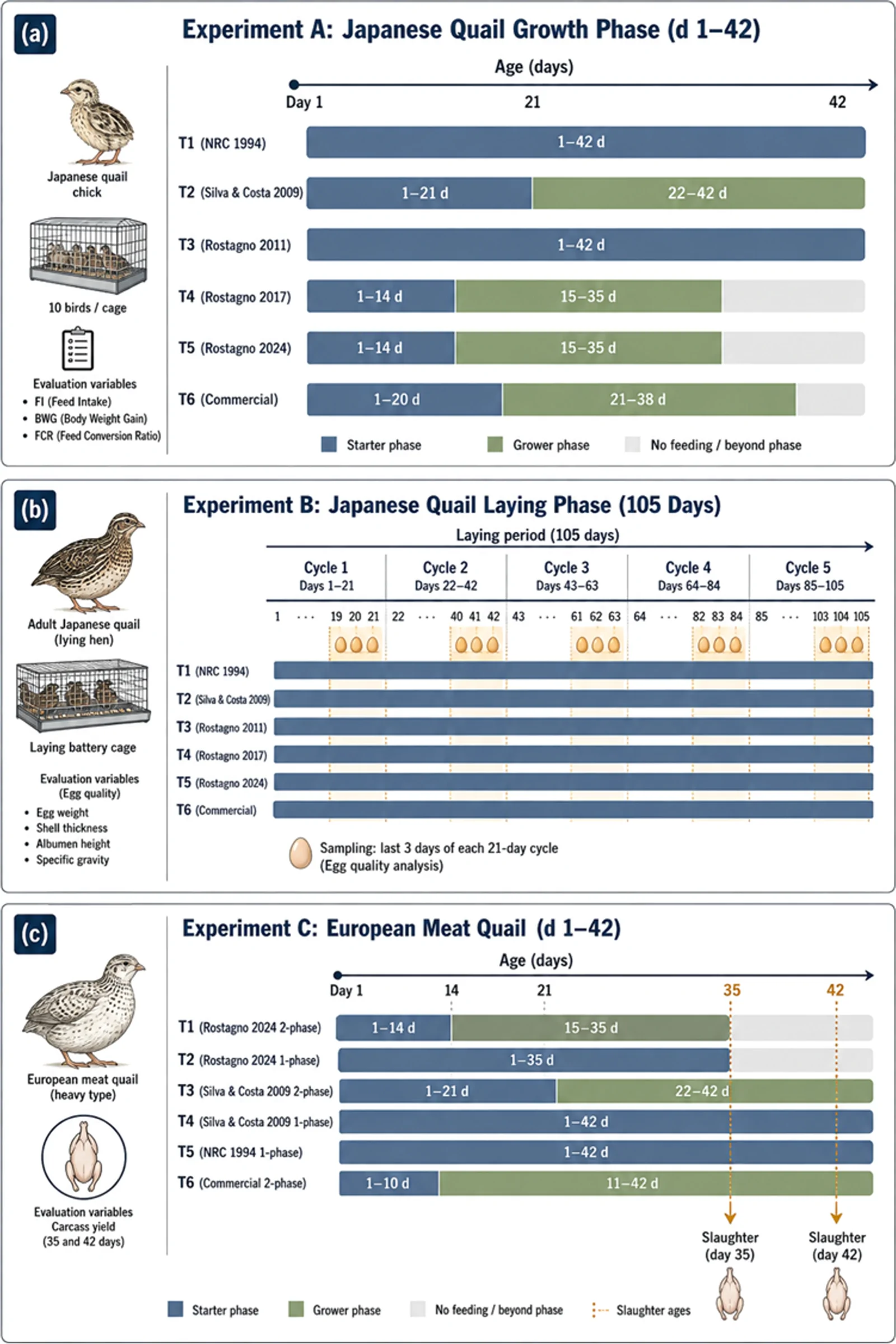
Schematic representation of the experimental designs, feeding programs, and evaluation timelines for Experiments A, B, and C. (a) Experiment A: Evaluation of single-phase and multi-phase nutritional plans for growing Japanese quails (0 to 42 d). (b) Experiment B: Evaluation of nutritional programs for laying Japanese quails across five 21-d cycles (105 d total), highlighting the egg quality sampling periods during the last 3 d of each cycle. (c) Experiment C: Comparison of single-phase versus two-phase feeding programs and slaughter ages (35 vs. 42 d) in growing European meat quails. All treatments used 10 replicates of 10 birds per cage.

Experiment B (Table 2 and Fig. 1.) – Japanese quail (laying phase): A total of 600 quail at 15 weeks of age with an average initial egg production rate of 74.48 ± 8.52 % were used, distributed into six treatments, with 10 replicates of 10 birds each. The treatments were as follows: T1 – NRC (1994); T2 – Silva and Costa (2009); T3 – Rostagno et al. (2011); T4 – Rostagno et al. (2017); T5 – Rostagno et al. (2024); and T6 – Commercial Manual. The experiment lasted 105 d, divided into five 21-d cycles.

**Table 2:** Nutritional requirements of laying Japanese quails.

Ingredients	Composition of diets (g/kg)					
	T1*	T2	T3	T4	T5	T6
Corn (7.88%)	594.79	589.08	564.78	511.10	566.87	231.08
Soybean meal	286.45	311.17	298.75	331.61	338.09	327.47
Wheat bran	12.07	—	36.26	47.68	—	253.11
Soybean oil	13.17	5.35	10.69	20.01	17.89	90.36
Limestone	70.28	68.94	67.46	66.93	54.44	74.71
Dicalcium phosphate	11.53	11.26	10.20	12.23	13.24	13.69
Salt	3.65	3.40	3.34	5.47	3.47	3.93
L-Lysine	1.793	2.824	1.965	0.247	0.699	0.978
DL-Methionine	3.065	4.147	3.351	1.512	1.466	1.460
L-Threonine	—	0.623	—	—-	0.642	—
Adsorbent	0.10	0.10	0.10	0.10	0.10	0.10
Antioxidant	0.10	0.10	0.10	0.10	0.10	0.10
Mineral Premix	1.50	1.50	1.50	1.50	1.50	1.50
Vitamin Premix	1.50	1.50	1.50	1.50	1.50	1.50
Total	1000.0	1000.0	1000.0	1000.0	1000.0	1000.0
Chemical composition (g/kg)						
Nutrientes	T1	T2	T3	T4	T5*	T6
Crude protein	180.00	180.92	180.71	200.00	200.00	200.80
Metab. energy (MJ/kg)	11.92	11.72	11.72	11.72	12.13	11.84
Crude fiber	29.83	30.96	29.70	30.04	31.79	22.74
Calcium	30.40	29.90	29.09	29.50	25.00	33.00
Available phosphorus	3.15	3.09	3.03	3.50	3.50	4.60
Sodium	1.57	1.47	1.45	2.30	1.50	1.70
Chloride	2.87	2.71	2.67	3.92	2.74	2.89
Potassium	7.28	7.58	7.67	8.23	8.00	9.52
Digestible lysine	9.93	11.49	10.45	9.50	10.00	10.50
Digestible methionine	5.65	6.83	6.00	4.31	4.30	4.17
Digestible M + C	8.14	9.42	8.57	7.00	7.00	6.90
Digestible threonine	6.06	7.01	6.27	6.70	7.40	6.70
Digestible tryptophan	1.97	2.09	2.07	2.25	2.23	2.43
Digestible valine	7.63	8.03	7.89	8.41	8.48	8.43
Digestible isoleucine	6.99	7.42	7.26	7.82	7.89	7.91

T1 - NRC (1994); T2 - Silva and Costa (2009); T3 - Rostagno et al. (2011); T4 - Rostagno et al. (2017); T5 - Rostagno et al. (2024); T6 - Commercial strain manual. *According to NRC recommendations expressed as total amino acids.

Experiment C (Table 3 and Fig. 1.) – European quails (meat-type): A total of 600 one-day-old quails straight-run, with an average initial body weight of 9.85 ± 0.14 g was used, distributed into six treatments, with 10 replicates of 10 birds each. The treatments were as follows: T1 – Rostagno et al. (2024), from 1 to 14 and 15 to 35 d; T2 – Rostagno et al. (2024), from 1 to 35 d; T3 – Silva and Costa (2009), from 1 to 21 and 22 to 42 d; T4 – Silva and Costa (2009), from 1 to 42 d; T5 – NRC (1994), from 1 to 42 d; and T6 – Commercial Manual, from 1 to 10 and 11 to 42 d of age. The variation in final slaughter age strictly followed the recommendations of each commercial guide or table tested.

**Table 3:** Nutritional requirements of European quails.

Ingredients	Composition of diets (g/kg)								
	T1		T2	T3		T4	T5	T6	
Corn (7.88%)	467.1	521.1	500.0	493.1	571.4	561.7	496.2	484.1	534.7
Soybean meal	458.7	406.5	427.3	454.3	376.5	397.4	475.7	477.3	425.7
Soybean oil	33.7	43.8	39.6	17.4	25.7	12.3	—	8.9	14.9
Limestone	10.2	7.6	8.6	11.2	9.3	9.8	1.3	10.2	7.6
Dicalcium phosphate	12.9	10.9	11.7	10.5	8.5	9.3	17.3	12.6	10.6
Salt	3.93	3.44	3.64	3.93	3.44	3.69	3.41	3.92	3.43
L-Lysine	1.21	0.22	0.62	0.96	—	—	—	—	—
DL-Methionine	4.92	2.35	3.38	3.74	2.0	2.69	3.08	—	—
L-Threonine	3.7	1.1	2.1	1.9	0.2	—	—	—	—
L-Valine	0.7	—	—	—	—	—	—	—	—
Mineral premix	1.5	1.5	1.5	1.5	1.5	1.5	1.5	1.5	1.5
Vitamin premix	1.5	1.5	1.5	1.5	1.5	1.5	1.5	1.5	1.5
Total	1000	1000	1000	1000	1000	1000	1000	1000	1000
Chemical composition (g/kg)									
Nutrients	T1		T2	T3		T4	T5*	T6	
Crude protein	250	230	238	250	220	230	260	260	240
Metab. energy (MJ/kg)	12.41	13.02	12.78	12.13	12.76	12.34	11.72	11.92	12.34
Crude fiber	27.8	26.5	27.0	28.0	26.0	26.8	29.0	28.9	27.5
Calcium	8.71	7.05	7.71	8.50	7.00	7.50	6.50	8.71	7.05
Available phosphorus	3.63	3.18	3.36	3.20	2.70	2.90	4.50	3.63	3.18
Sodium	1.70	1.50	1.58	1.70	1.50	1.60	1.50	1.70	1.50
Chloride	2.99	2.72	2.83	3.01	2.76	2.90	2.72	3.01	2.74
Potassium	9.89	9.11	9.42	9.89	8.72	9.07	10.28	10.28	9.50
Digestible lysine	14.01	11.78	12.67	13.70	10.20	11.40	13.31	13.33	12.09
Digestible methionine	8.19	5.42	6.53	7.03	4.98	5.77	6.50	3.45	3.23
Digestible M + C	11.55	8.55	9.75	10.40	8.00	8.90	10.00	6.94	6.49
Digestible threonine	12.20	8.96	10.26	10.40	7.80	7.00	8.90	8.90	8.23
Digestible valine	11.31	9.71	10.05	10.57	9.26	9.65	11.00	11.00	10.13

T1 - Rostagno et al. (2024) – 1 to 14 and 15 to 35 days; T2 - Rostagno et al. (2024) – 1 to 35 days; T3 - Silva and Costa (2009) – 1 to 21 and 22 to 42 days; T4 - Silva and Costa (2009) – 1 to 42 days; T5 - NRC (1994) – 1 to 42 days; T6 - Commercial Manual – 1 to 10 and 11 to 42 days of age. *According to NRC recommendations expressed as total amino acids.

### Experimental variables

#### Performance

For Experiments A and C, the variables evaluated weekly were feed intake, body weight gain, feed conversion ratio, and mortality. Mortality was monitored and recorded daily, and feed intake and feed conversion ratio were adjusted using the bird-day weighted average method as described by Sakomura and Rostagno (2016).

At the end of Experiment C, 2 birds per replicate (20 birds per treatment) representing the mean plot body weight were selected for carcass yield evaluation. Following an 8-hour pre-slaughter feed withdrawal period with free access to water, birds were weighed, stunned by cervical dislocation, bled, scalded, plucked, and eviscerated. Carcass yield was determined relative to live weight at slaughter. Considering that the commercial quail industry markets whole carcasses and does not commercialize cut-up parts, evaluation focused on whole eviscerated carcass yield and abdominal fat percentage expressed relative to eviscerated carcass weight.

For Experiment B (egg-production phase), the evaluated variables were feed intake, egg production, egg weight, egg mass, and feed conversion ratio per egg mass and per dozen eggs. Additionally, for Experiment B, age at first egg and body weight change between the beginning and end of the experimental period were recorded.

Feed intake was determined by weighing feed refusals and subtracting them from the amount of feed initially provided throughout the experimental period. At the end of each 21-d period, feed intake was divided by the number of birds per treatment and by the number of days to calculate the average grams of feed consumed per bird per day adjusted for mortality.

Egg production (EP, %) was determined by recording the number of eggs produced daily, including broken, cracked, and abnormal eggs (e.g., soft-shelled eggs). This value represented the average EP of birds during each period. Egg mass (EM, g) was calculated by multiplying the average egg weight by the number of eggs produced during the experimental period.

Feed conversion ratio per egg mass was calculated by dividing total feed intake (g) by total egg mass produced. Feed conversion per dozen eggs was calculated by dividing total feed intake (g) by the number of dozens of eggs produced. Body weight variation (g/bird) was obtained by weighing the birds at the beginning and at the end of the experimental period.

#### Egg quality

Egg quality analyses were performed during the last three days of each 21-d period. Three eggs with average weight from each replicate were collected, individually identified, and weighed using an analytical balance. To ensure unbiased evaluation, physical and shell analyses were conducted under a single-blind arrangement, in which evaluators were unaware of treatment identities. Subsequently, eggs were broken onto a flat surface to measure albumen height (mm) using a depth micrometer (model S-8400, Ames®, Boston, MA, USA), followed by recording yolk weight. The eggshells were then washed, dried in a forced-ventilation oven at 45°C for 48 h, and weighed. Albumen weight was calculated by difference (egg weight − shell weight − yolk weight). Shell thickness was measured using a digital micrometer at three equidistant points along the equatorial region of the eggshell.

Eggshells were dried in a forced-air oven at 45°C for 48 h and subsequently weighed. Albumen weight was obtained by difference (egg weight − shell weight − yolk weight). Eggshell thickness was measured using a digital micrometer at three equidistant points in the equatorial region of the shell, and the arithmetic mean was used as the representative value.

Specific gravity was determined using the saline flotation method (Hamilton, 1982) by immersing eggs in sodium chloride (NaCl) solutions with densities ranging from 1.0700 to 1.0975 g/cm³, in increments of 0.0025 g/cm³. The density of each solution was regularly verified using a petroleum densimeter. The specific gravity of each egg was recorded as the density of the solution in which the egg floated.

### Statistical analysis

Data were evaluated for normality of errors using the Shapiro–Wilk test (Shapiro and Wilk, 1965) and for homogeneity of variances using Levene's test (Levene, 1960). The assumptions of normality and homoscedasticity were met for all variables analyzed.

For variables evaluated on a consolidated basis over the experimental period, data were subjected to one-way analysis of variance (ANOVA) according to the following statistical model:

$$Y_{ij}=\mu +T_{i}+e_{ij}$$

where Yᵢⱼ is the observed value of the response variable in replicate j receiving treatment i; μ is the overall mean; Tᵢ is the fixed effect of the nutritional treatment i; and eᵢⱼ is the random experimental error.

In Experiment B, egg quality variables were evaluated based on the cumulative data collected throughout the experimental period, comprising 450 eggs per treatment (45 eggs per replicate). Thus, the analysis focused on the overall response of the experimental units to the nutritional programs rather than on individual collection cycles.

When significant differences were detected by ANOVA, treatment means were compared using Tukey's test (Tukey, 1949) at a 5% significance level (P < 0.05). All statistical analyses were performed using SAS OnDemand for Academics (SAS Institute Inc., Cary, NC, USA).

## Results and discussion

### Experiment A – Japanese quail during the growing phase

For Japanese quail during the growing phase (Table 4), nutritional plans based on the recommendations of NRC (1994), Rostagno et al. (2011), and Silva and Costa (2009) resulted in higher feed intake and greater weight gain compared with the more recent recommendations of the Brazilian Tables for Poultry and Swine (Rostagno et al., 2017; Rostagno et al., 2024) and the commercial strain manual (P<0.001).

**Table 4:** Growth performance of Japanese quails subjected to different nutritional plans during the growing period.

	T1	T2	T3	T4	T5	T6	*P-value*	CV
FI	14.81a	14.20b	15.17a	13.12c	12.90c	14.25b	<0.001	4.36
WG	4.17a	4.19a	4.19a	3.75c	3.83c	3.97b	<0.001	2.96
FCR	3.53ab	3.35b	3.62a	3.41b	3.37b	3.59a	0.0455	5.91
AFE	38.14	37.22	37.37	37.71	36.87	37.28	0.4593	3.27

T1 - NRC (1994) - 1 to 42 days; T2 - Silva and Costa (2009) - 1 to 21 and 22 to 42 days; T3 - Rostagno et al. (2011) - 1 to 42 days; T4 - Rostagno et al. (2017) - 1 to 14 and 15 to 35 days; T5 - Rostagno et al. (2024) - 1 to 14 and 15 to 35 days; T6 - Commercial strain manual - 1 to 20 and 21 to 38 days of age. FI = feed intake (g/quail/day); WG = weight gain (g/quail/day); FCR = feed conversion ratio (g/g); AFE = age at first egg (days).

Feed conversion ratio was apparently similar among treatments, with the lowest values observed for the recommendations proposed by Silva and Costa (2009), Rostagno et al. (2017), and Rostagno et al. (2024) (P=0.0455).

The diet formulated based on the recommendations of T1 – NRC (1994) presented the lowest energy:protein ratio (Table 5), using a single diet throughout the entire growth period. In contrast, the nutritional programs T2 – Silva and Costa (2009), T4 – Rostagno et al. (2017), T5 – Rostagno et al. (2024), and T6 – Commercial Manual adopted a multi-phase feeding strategy, characterized by lower energy:protein ratios in the initial phase and a progressive increase in this ratio as birds aged.

**Table 5:** Nutritional ratios of experimental diets for growing Japanese quails subjected to different nutritional plans.

Nutrients	T1*	T2		T3	T4		T5		T6	
Crude protein (g/kg)	240	250	220	220	240	230	250	250	230	220
Metab. energy (MJ/kg)	12.13	12.13	12.76	12.13	12.13	12.13	11.92	11.92	12.44	12.34
Nutritional Ratios										
ME:CP	50.5	48.5	58.0	55.1	49.8	52.7	46.4	47.3	53.4	55.3
Digestible Met:Lys	0.326	0.388	0.401	0.408	0.334	0.329	0.433	0.374	0.404	0.410
Digestible Met+Cys:Lys	0.577	0.672	0.678	0.679	0.601	0.603	0.680	0.670	0.684	0.686
Digestible Thr:Lys	0.785	0.731	0.752	0.705	0.675	0.689	0.670	0.783	0.649	0.693
Calcium:Phosphorus	2.67	2.66	2.50	2.40	2.13	2.13	1.73	1.96	1.96	2.04

T1 - NRC (1994) – 1 to 42 days; T2 - Silva and Costa (2009) – 1 to 21 and 22 to 42 days; T3 - Rostagno et al. (2011) – 1 to 42 days; T4 - Rostagno et al. (2017) – 1 to 14 and 15 to 35 days; T5 - Rostagno et al. (2024) – 1 to 14 and 15 to 35 days; T6 - Commercial strain manual – 1 to 20 and 21 to 38 days of age. *According to NRC recommendations expressed as total amino acids.

The T3 – Rostagno et al. (2011) plan, although it also used a single diet throughout the experimental period, showed a higher energy:protein ratio than that observed for NRC (1994). The protein content of the diets recommended by T2 – Silva and Costa (2009), T4 – Rostagno et al. (2017), T5 – Rostagno et al. (2024), and T6 – Commercial Manual decreased by 12, 5.54, 1.95, and 5.11%, respectively, from the first to the second diet (Table 5).

Feed intake and body weight gain (Table 4) appear to be directly related to differences in nutrient ratios among diets (Table 5), particularly metabolizable energy:crude protein (ME:CP). In general, diets with lower ME:CP ratios (Table 5) tend to increase feed intake, as birds adjust consumption to meet their energy requirements (Silva et al., 2001; Nascimento et al., 2004; Mosaad and Iben, 2009; Musigwa et al., 2024), especially in nutritional programs using a single diet throughout the growth period.

These assumptions may explain the higher feed intake observed (Table 4) in birds fed the T1 – NRC (1994) diet, since dietary energy content is one of the main regulators of voluntary feed intake (Macari et al., 1994; Leeson and Summers, 2001; Forbes, 2007; Macari and Maiorka, 2017), and in this treatment, an energy value of 12.13 MJ/kg was used throughout the entire quail growth period.

Contradictorily, the diet formulated based on T3 – Rostagno et al. (2011) recommendations also presented the highest energy:protein ratio; however, birds subjected to this treatment showed higher feed intake. This result may be associated with the single nutritional plan adopted throughout the entire growth period, without the use of multi-phase feeding, and the diet presenting the lowest protein and amino acid content among treatments. Thus, it is likely that the diet did not adequately follow the changes in nutritional requirements of the birds throughout development, leading to higher voluntary feed intake in an attempt to compensate for possible nutritional imbalances, especially related to amino acid and energy supply.

Quail fed diets formulated according to T2 – Silva and Costa (2009) and T6 – Commercial Manual showed similar feed intake (Table 4), and higher than those fed diets based on T4 – Rostagno et al. (2017) and T5 – Rostagno et al. (2024), which did not differ from each other. When observing T2 and T6 diets, protein content decreased by 12 and 5.11%, respectively, compared with T4 and T5 diets, where reductions were 5.54 and 1.95%, respectively. This indicates a better balance between protein and energy in the latter diets, whereas diets with greater nutritional imbalance (T2 – Silva and Costa, 2009 and T6 – Commercial Manual) led to increased voluntary feed intake as a compensatory mechanism (Macari et al., 1994; Leeson and Summers, 2001; Forbes, 2007; Macari and Maiorka, 2017).

These findings highlight the need for proper adjustment of protein and energy levels during quail growth, where early phases require greater amino acid and mineral support, and later phases require greater energy supply. Vieira et al. (2026) suggest that Japanese quail should be fed three diets during the growth phase (1–18, 19–29, and 30–36 d of age). Similarly, recent evidence indicates that mineral requirements also vary between growth phases in Japanese quails, with separate calcium and available phosphorus requirements estimated for the starter (1–14 d) and grower (15–35 d) periods (Vieira et al., 2025). Together, these findings support the use of phase-specific nutritional programs to better align nutrient supply with the changing requirements of growing quails.

The lowest calcium:phosphorus and amino acid:lysine ratios (Table 5) were observed in diets (Table 1) formulated based on T4 – Rostagno et al. (2017) and T5 – Rostagno et al. (2024) recommendations. Although both recommendations presented lower ratios, the diet proposed by T5 – Rostagno et al. (2024) showed the highest amino acid density.

The lowest feed conversion ratio, intermediate body weight gain (P<0.001) among treatments, lower feed intake, and earlier age at first egg were observed in T5 – Rostagno et al. (2024). According to Vieira et al. (2026), growing Japanese quail have a higher demand for protein and amino acids during the first 18 d of age; the Commercial Manual recommendation meets this requirement only for the first 10 d, whereas Rostagno et al. (2024) recommendations cover the first 14 d, allowing for a better adjustment to nutritional requirements.

Age at first egg (Table 4) was not significantly affected by treatments, indicating that, despite differences in the chemical composition of the experimental diets, quail were able to adjust feed intake and nutrient utilization to maintain reproductive development (Omidiwura et al., 2016; Ashour et al., 2024; Reda et al., 2025). These results suggest that the nutritional levels used, although different, remained within a range that allowed birds to meet physiological demands for maintenance and growth without impairing the onset of reproductive activity, even with differences observed in feed intake and body weight gain (Table 4).

Age at first egg appears to be strongly associated with the genetic potential of the birds, especially due to advances in genetic selection of modern strains, making this parameter less sensitive to moderate variations in dietary composition. Tavárez and Santos (2016) stated that 80–90% of improvements observed in the performance of modern poultry are attributed to genetic selection, whereas nutrition and management are essential to allow the expression of this genetic potential.

Results from Havenstein et al. (2003), comparing broiler strains from 1957 and 2001 fed diets representative of different periods, also demonstrated that the greatest advances in performance and carcass yield were primarily due to genetic progress, while nutritional improvements played a complementary role in maximizing this potential.

Thus, even under moderate changes in nutritional levels, birds may exhibit compensatory mechanisms in feed intake and nutrient utilization, preserving key reproductive traits such as sexual maturity and onset of lay. Notably, more nutritionally balanced diets (T5 – Rostagno et al. (2024) – 1 to 14 and 15 to 35 d) tended to favor earlier age at first egg, likely due to improved metabolic and body support for the onset of laying.

The findings of the present study reinforce the hypothesis that multi-phase nutritional programs and formulations better aligned with the ideal protein concept (T5 – Rostagno et al. (2024) – 1 to 14 and 15 to 35 d) promote greater nutrient utilization efficiency. Although the recommendations of Silva and Costa (2009) also adopt a multi-phase feeding strategy, the higher feed intake observed suggests that the established nutritional levels may not adequately meet the current requirements of the birds, leading to increased voluntary feed intake as a compensatory mechanism, since these diets must cover a longer growth period in the initial phase (1–22 d of age) compared with Rostagno et al. (2024), in which a single diet is used only during the first 14 d.

Vieira et al. (2026), using nonlinear models of growth and nutrient deposition in Japanese quail, suggested that nutritional programs with a greater number of feeding phases may provide a better adjustment between nutrient supply and requirements throughout growth, thereby improving nutrient utilization efficiency and bird performance.

### Experiment B – Japanese Quail during the laying phase

The greatest eggshell thickness (ET) was observed in birds fed (Table 6) the Rostagno et al. (2017) plan (T4), whereas the lowest value was recorded for the Commercial Manual (T6) (P=0.0494). For specific gravity (SG), the highest value was observed in the treatment based on Rostagno et al. (2024) (T5), differing from NRC (1994) (T1), which showed the lowest value (P=0.0205). Eggshell percentage (EP) was also higher in birds fed the Rostagno et al. (2024), diet, whereas the lowest values were observed for birds fed diets based on T1 – NRC (1994), T2 – Silva and Costa (2009), T3 – Rostagno et al. (2011), and T6 – Commercial strain manual (P=0.0184).

**Table 6:** Egg quality traits of Japanese quails subjected to different nutritional plans during the laying period.

Variáveis	T1	T2	T3	T4	T5	T6	*P-value*	CV
ET	0.220ab	0.220ab	0.220ab	0.230a	0.220ab	0.210b	0.0494	11.55
SG	1.050b	1.060ab	1.060ab	1.060ab	1.070a	1.060ab	0.0205	3.97
EP	8.46b	8.53b	8.46b	8.73ab	8.90a	8.48b	0.0184	12.53
YP	29.80b	30.09b	31.42a	30.46ab	30.19b	30.55ab	0.0401	11.96
AP	61.24	61.38	60.13	60.8	61.35	61.05	0.2316	6.33

T1 - NRC (1994); T2 - Silva and Costa (2009); T3 - Rostagno et al. (2011); T4 - Rostagno et al. (2017); T5 - Rostagno et al. (2024); T6 - Commercial strain manual. ET = eggshell thickness (mm); SG = specific gravity (g/cm³); EP = eggshell percentage (%); YP = yolk percentage (%); AP = albumen percentage (%).

The highest yolk percentage (YP) was observed in birds fed the Rostagno et al. (2011) plan (T3), differing from T1 – NRC (1994), T2 – Silva and Costa (2009), and T5 – Rostagno et al. (2024) (P=0.0401). No significant differences were observed for albumen percentage (AP) among the evaluated treatments (P=0.2316).

For the productive performance of laying Japanese quail (Table 7), the different nutritional plans significantly affected egg production (EPr) (P=0.0006), egg mass (EM) (P=0.0003), and feed conversion ratio per egg mass (FCR_EM_) (P=0.0022) and per dozen eggs (FCR_Dz_) (P=0.0019). Birds fed the Rostagno et al. (2024) plan (T5) showed the highest egg production and egg mass, whereas the lowest values were observed in the Commercial Manual (T6).

**Table 7:** Productive performance of Japanese quails subjected to different nutritional plans during the laying period.

Variáveis	T1	T2	T3	T4	T5	T6	*P-value*	CV
EPr	77.32b	82.46b	82.83b	84.95ab	89.88a	68.06c	0.0006	9.23
EM	8.49b	9.04ab	9.05ab	9.15ab	9.51a	7.36c	0.0003	9.25
FCR_EM_	2.83a	2.69a	2.91a	3.14a	2.55ab	2.15b	0.0022	13.52
FCR_Dz_	0.370a	0.360a	0.380a	0.410a	0.300ab	0.280b	0.0019	13.68

T1 - NRC (1994); T2 - Silva and Costa (2009); T3 - Rostagno et al. (2011); T4 - Rostagno et al. (2017); T5 - Rostagno et al. (2024); T6 - Commercial strain manual. EPr = egg production (%); EM = egg mass (g/quail/day); FCR_EM_ = feed conversion ratio per egg mass (kg/kg); FCR_Dz_ = feed conversion ratio per dozen eggs (kg/dozen).

Regarding feed conversion ratio per egg mass (FCREM) and per dozen eggs (FCRDz), the lowest values were observed in T6 – Commercial Manual, differing from T1 – NRC (1994), T2 – Silva and Costa (2009), T3 – Rostagno et al. (2011), and T4 – Rostagno et al. (2017).

The results of egg quality and productive performance of laying Japanese quail indicate that the different nutritional programs influenced both production efficiency and egg physical characteristics (Table 6, Table 7). These responses may be associated with differences in dietary protein and energy density, as well as in the balance among calcium, phosphorus, and digestible amino acids (Table 8). This interpretation is consistent with recent findings in laying Japanese quails, in which dietary protein and metabolizable energy interacted to affect productive performance and egg quality. In that study, the combination of 20% crude protein and 2,900 kcal/kg ME resulted in greater egg mass, demonstrating that productive responses depend on the balance between protein and energy rather than on either nutrient concentration alone (Ashour et al., 2024).

**Table 8:** Nutritional ratios and amino acid:lysine relationships of the experimental diets fed to Japanese quails during the laying phase.

Nutrients	T1*	T2	T3	T4	T5	T6
Crude protein (g/kg)	180	180.9	180.7	200	200	200.8
Metabolizable energy (MJ/kg)	11.92	11.72	11.72	11.72	12.13	11.84
Nutritional Ratios						
ME:CP	66.2	64.8	64.9	58.6	60.7	59.0
Digestible Lys:CP	0.552	0.607	0.559	0.475	0.500	0.505
Digestible Met+Cys:Lys	0.820	0.820	0.820	0.737	0.700	0.657
Digestible Thr:Lys	0.610	0.610	0.600	0.705	0.740	0.638
Digestible Val:Lys	0.768	0.699	0.755	0.885	0.848	0.803
Digestible Ile:Lys	0.704	0.646	0.695	0.823	0.789	0.753
Calcium:phosphorus	9.65	9.68	9.60	8.43	7.14	7.17

T1 - NRC (1994); T2 - Silva and Costa (2009); T3 - Rostagno et al. (2011); T4 - Rostagno et al. (2017); T5 - Rostagno et al. (2024); T6 - Commercial strain manual. *According to NRC recommendations expressed as total amino acids.

The nutritional programs differed substantially in crude protein and metabolizable energy concentrations. The NRC (1994), Silva and Costa (2009), and Rostagno et al. (2011) programs (T1, T2, and T3, respectively) provided approximately 180 g/kg of crude protein, whereas the Rostagno et al. (2017), Rostagno et al. (2024), and Commercial strain manual programs (T4, T5, and T6, respectively) provided approximately 200 g/kg. Consequently, the latter programs presented lower ME ratios, particularly T4 (58.6), T6 (59.0), and T5 (60.7), compared with T1 (66.2), T3 (64.9), and T2 (64.8). This indicates a greater protein supply relative to dietary energy in T4, T5, and T6, which may have influenced nutrient partitioning and productive responses during the laying period.

The differences in calcium ratios were also pronounced among the nutritional programs, ranging from 7.14 in T5 – Rostagno et al. (2024) to 9.68 in T2 – Silva and Costa (2009). The highest ratios were observed in T2 – Silva and Costa (2009), T1 – NRC (1994), and T3 – Rostagno et al. (2011), whereas T4 – Rostagno et al. (2017), T5 – Rostagno et al. (2024), and T6 – Commercial strain manual presented lower ratios. However, the highest specific gravity and eggshell percentage were observed in T5 – Rostagno et al. (2024), which had the lowest calcium ratio among the evaluated programs. Therefore, the present results do not support a direct association between a higher calcium ratio and improved eggshell quality. Rather, eggshell quality may have resulted from the interaction among calcium and phosphorus supply, energy and protein density, and the balance of other nutrients involved in eggshell formation and mineralization (Roland, 1986; Keshavarz and Nakajima, 1993).

The greatest eggshell thickness was observed in T4 – Rostagno et al. (2017), which presented a relatively low ME ratio (58.6) and 200 g/kg of crude protein. In contrast, the lowest eggshell thickness was observed in T6 – Commercial strain manual, despite its similarly high crude protein concentration (200.8 g/kg) and low ME ratio (59.0). Thus, the differences in eggshell thickness cannot be explained solely by crude protein concentration or ME ratio, suggesting that the balance among mineral and amino acid nutrients may also have contributed to the observed response. The organic matrix of the eggshell plays an important role in nucleation, mineral deposition, and organization of calcium carbonate crystals during eggshell biomineralization (Arias and Fernandez, 2001; Nys et al., 2004).

The higher specific gravity and eggshell percentage observed in T5 – Rostagno et al. (2024) indicate that this nutritional program provided a favorable combination of nutrients for external egg quality. This treatment contained 200 g/kg of crude protein, an ME ratio of 60.7, and a digestible Lys ratio of 0.500, together with relatively high digestible threonine, valine, and isoleucine relationships with lysine (0.740, 0.848, and 0.789, respectively). Although these values cannot be used to establish a direct causal relationship with eggshell quality, they illustrate the different nutritional profiles provided by the evaluated programs and reinforce the importance of considering the overall nutrient balance rather than isolated nutrient concentrations.

The higher egg production and egg mass observed in T5 – Rostagno et al. (2024) may also reflect the greater alignment between nutrient supply and the requirements of laying Japanese quail. This program provided 200 g/kg of crude protein and the highest digestible Lys ratio among the treatments (0.500, except for T2 with 0.607), together with a relatively low ME ratio (60.7). Adequate dietary protein and essential amino acid supply are important for maintaining egg production and egg mass in laying Japanese quail (Kaur et al., 2017; El-Hindawy et al., 2021; Ashour et al., 2024). However, because T6 – Commercial strain manual also provided a high crude protein concentration but resulted in the lowest egg production and egg mass, these responses likely reflect the combined effects of amino acid balance, energy supply, and other nutritional characteristics rather than crude protein concentration alone.

The higher yolk percentage observed in birds fed the Rostagno et al. (2011) program (T3) may be related to its overall energy and amino acid profile. T3 presented an ME ratio of 64.9, which was relatively high among the evaluated diets, although lower than that of T1 – NRC (1994) (66.2). Diets with relatively greater energy availability in relation to protein may favor lipid deposition and, consequently, influence yolk deposition. Nevertheless, the difference in yolk percentage observed among treatments should be interpreted cautiously, as yolk formation is affected by the interaction among energy intake, protein and amino acid supply, lipid metabolism, and nutrient partitioning.

The digestible Met+Cys ratio may also have contributed to differences in egg composition. The highest ratio (0.820) was observed in T1 – NRC (1994), T2 – Silva and Costa (2009), and T3 – Rostagno et al. (2011), whereas lower values were observed in T4 – Rostagno et al. (2017), T5 – Rostagno et al. (2024), and T6 – Commercial strain manual. Sulfur amino acids, particularly methionine, participate in methyl-group metabolism and in the synthesis of compounds such as choline and phosphatidylcholine, which are involved in hepatic lipid metabolism and the formation and secretion of very-low-density lipoproteins (VLDL), responsible for transporting lipids to the developing ovarian follicles (Dong et al., 2019; Abd El-Ghany et al., 2022). Therefore, the relatively high Met+Cys ratios in T1, T2, and T3 may have contributed to differences in nutrient partitioning and yolk deposition, although the present experiment does not allow this mechanism to be established directly.

Overall, the different nutritional programs affected both productive performance and egg quality of Japanese quail during the laying phase. The results indicate that these responses were not determined by a single nutritional factor, but rather by the combined balance among energy, protein, calcium, phosphorus, and digestible amino acids. In particular, the Rostagno et al. (2024) program (T5) resulted in the highest egg production, egg mass, specific gravity, and eggshell percentage, whereas the Rostagno et al. (2017) program (T4) resulted in the greatest eggshell thickness. These findings highlight the importance of evaluating complete nutritional profiles when comparing feeding programs for laying Japanese quail, rather than attributing productive or egg-quality responses to isolated nutrient ratios.

### Experiment C – European Quail during the growing phase and carcass evaluation

In the first week (Table 9), European quail fed the T5 – NRC (1994) plan showed greater weight gain and higher body weight, whereas the lowest values were observed for T2 – Rostagno et al. (2024) using a single program from 1 to 35 d and for T6 – Commercial Manual (P<0.01). Also in the first week, better feed conversion ratios were observed in treatments based on T3 – Silva and Costa (2009), using a two-diet program, and T5 – NRC (1994) (P<0.01). In the second week, T5 – NRC (1994) maintained higher feed intake (P=0.021) and body weight but showed poorer feed conversion compared with the multi-phase plan of Rostagno et al. (2024).

**Table 9:** Weekly productive performance of European quails subjected to different nutritional plans from 1 to 6 weeks of age.

Variables	T1	T2	T3	T4	T5	T6	*P-value*	CV
Week 1								
FI	5.63	5.74	5.51	5.85	5.76	5.40	0.082	6.52
WG	3.59bc	3.45c	3.8ab	3.57bc	3.88a	3.49c	<.0.01	5.64
FCR	1.58ab	1.67a	1.45b	1.64a	1.48b	1.55ab	<.0.01	7.27
LW	35.27bc	33.97c	36.45ab	34.86bc	37.02a	34.29c	<.0.01	3.66
Week 2								
FI	13.03ab	12.89b	13.78ab	13.49ab	14.21a	13.4ab	0.021	6.67
WG	6.74	6.46	6.39	6.14	6.72	6.45	0.158	8.46
FCR	1.95b	2.01ab	2.17ab	2.21a	2.12ab	2.08ab	0.027	9.02
LW	82.44ab	79.17ab	81.83ab	77.83b	84.05a	79.46ab	0.010	5.01
Week 3								
FI	16.75c	17.75bc	18.62ab	18.26ab	19.2a	18.2ab	<.0.01	5.98
WG	6.97b	8.45a	8.58a	8.33a	8.98a	8.77a	<.0.01	7.57
FCR	2.59a	2.12b	2.18ab	2.2ab	2.14ab	2.08b	0.025	5.92
LW	133.3c	138.31bc	141.88ab	137.4bc	146.92a	140.83abc	<.0.01	4.34
Week 4								
FI	22.78ab	21.95b	23.22ab	22.42ab	23.51a	22.62ab	0.053	5.07
WG	8.55a	7.48ab	7.57ab	7.78ab	7.18ab	6.33b	<.0.01	5.81
FCR	2.75b	3.02b	3.1ab	2.89b	3.31ab	3.67a	<.0.01	5.43
LW	193.31abc	191.05bc	194.86ab	191.84abc	198.87a	186.92c	<.0.01	2.83
Week 5								
FI	28.52ab	26.65b	28.05ab	29.14a	28.84ab	28.73ab	0.033	6.15
WG	7.58a	5.86bc	5.36bc	5.82bc	4.58c	6.18ab	<.0.01	9.25
FCR	3.93c	4.62bc	5.27b	5.11b	6.47a	4.72bc	<.0.01	4.20
LW	249.86a	232.10b	232.37b	232.60b	230.94b	230.2b	<.0.01	5.37
Week 6								
FI	—	—	30.84	31.03	28.80	29.86	0.261	9.15
WG	—	—	4.19	4.32	4.02	4.42	0.778	8.40
FCR	—	—	7.55	7.49	7.35	7.09	0.911	6.47
LW	—	—	266.97	265.62	259.09	264.50	0.615	5.28

T1 - Rostagno et al. (2024) – 1 to 14 and 15 to 35 days; T2 - Rostagno et al. (2024) – 1 to 35 days; T3 - Silva and Costa (2009) – 1 to 21 and 22 to 42 days; T4 - Silva and Costa (2009) – 1 to 42 days; T5 - NRC (1994) – 1 to 42 days; T6 - Commercial Manual – 1 to 10 and 11 to 42 days of age. FI = feed intake (g/quail/day); WG = weight gain (g/quail/day); FCR = feed conversion ratio; LW = live weight (g/quail); P-value = probability value (%); coefficient of variation (%).

In the third week (Table 9), treatments T3 and T4 based on Silva and Costa (2009), T5 – NRC (1994), and T6 – Commercial Manual showed greater weight gain compared with T1 – Rostagno et al. (2024) (P<0.01). However, T1 and T2 (Rostagno et al., 2024) continued to show better feed conversion ratios across different weeks (P=0.025). In the fourth week, the highest body weight (P<0.01) was again observed in T5 – NRC (1994), whereas the Commercial Manual showed poorer feed conversion (P<0.01). In the fifth week, the multi-phase plan T1 – Rostagno et al. (2024) provided greater weight gain (P<0.01), better feed conversion (P<0.01), and higher final body weight (P<0.01) compared with most of the other treatments.

The results demonstrate that the effects of nutritional plans were more evident during the first four weeks of growth, a phase characterized by intense protein deposition, rapid muscle growth, and high metabolic demand. Initially, it was expected that the more recent recommendations of Rostagno et al. (2024), formulated based on digestible amino acids, the ideal protein concept, and multi-phase feeding, would provide consistent superiority across all evaluated productive variables. However, although this program showed better feed efficiency at different growth stages, the highest weight gains and initial body weights were observed in some older nutritional programs, such as NRC (1994) and Silva and Costa (2009).

These findings suggest that, despite advances in nutritional balancing and the adoption of multi-phase feeding programs, productive responses in quail may vary according to the genetic characteristics of the strains, growth potential, and the birds’ metabolic adaptability. Furthermore, classical nutritional recommendations often include higher absolute levels of crude protein, which may promote faster initial growth, although not necessarily resulting in better feed efficiency. Ribeiro et al. (2020) reported similar behavior in European quail, indicating that changes in protein and energy levels significantly affect growth curves.

On the other hand, the results obtained with Rostagno et al. (2024) reinforce the importance of continuously updating nutritional programs in response to genetic progress in modern commercial strains. Currently, new Japanese quail strains indicate the need for three dietary phases during growth (Vieira et al., 2026), and European quail may be following the same trend, given ongoing genetic improvements related to growth rate, feed efficiency, and productive potential.

Despite promoting greater initial growth, birds fed according to the T5 – NRC (1994) plan did not achieve the best feed conversion ratios. The Rostagno et al. (2024) programs showed higher feed efficiency in different weeks, possibly due to the more precise balance between digestible amino acids and metabolizable energy (Kidd and Anthony, 1997; Emmert and Baker, 1997; Vieira and Angel, 2012). Recent evidence in European quails also indicates that dietary energy density can modify feed intake and feed efficiency without necessarily producing proportional changes in carcass traits. Jesus et al. (2023) reported a quadratic response in feed efficiency to dietary metabolizable energy and estimated approximately 3,009 kcal/kg ME as adequate for European quails from 28 to 42 d of age. These findings further support the importance of balancing dietary energy with other nutrients rather than maximizing energy density alone.

Modern recommendations are based on the ideal protein concept, adjusting amino acid ratios to meet the physiological requirements of the birds and reducing excesses or imbalances. This approach improves metabolic utilization of amino acids, reduces energy losses associated with protein catabolism (Noblet and Henry, 1993; Emmans, 1994; MacLeod, 1997), decreases heat increment resulting from excess protein, and enhances feed efficiency (Musharaf and Latshaw, 1999; Vieira et al., 2018; Adhikari et al., 2025; Alabi and Adedokun, 2025). Additionally, Ghiasvand et al. (2025) reported that proper amino acid balance improves protein deposition and feed efficiency in meat-type quail.

When relating the nutritional profiles of the different feeding programs (Table 10) to the productive performance and carcass characteristics of European quail (Table 11), it is evident that differences in energy:protein ratios and amino acid balance directly influenced the birds’ productive responses.

**Table 10:** Energy-to-protein ratio, calcium-to-available phosphorus ratio, and amino acid relationships relative to lysine in experimental diets formulated.

Nutrients	T1		T2	T3		T4	T5*	T6	
Crude protein g/kg	250		230	238		250	220	230	
Metab. energy (MJ/kg)	12.41		13.02	12.78		12.13	12.76	12.34	
Nutritional Ratios									
ME:CP	49.64	56.61	53.70	48.52	58.00	53.65	45.08	45.85	51.42
Digestible Lys:CP	5.60	5.12	5.32	5.48	4.64	4.96	5.12	5.13	5.04
Digestible Met+Cys:Lys	82.44	72.58	76.95	75.91	78.43	78.07	75.13	52.06	53.68
Digestible Thr:Lys	87.08	76.06	80.98	75.91	76.47	61.40	66.87	66.77	68.07
Digestible Val:Lys	80.73	82.43	79.32	77.15	90.78	84.65	82.64	82.52	83.79
Calcium:Phosphorus	2.40	2.22	2.29	2.66	2.59	2.59	1.44	2.40	2.22

T1 - Rostagno et al. (2024) – 1 to 14 and 15 to 35 days; T2 - Rostagno et al. (2024) – 1 to 35 days; T3 - Silva and Costa (2009) – 1 to 21 and 22 to 42 days; T4 - Silva and Costa (2009) – 1 to 42 days; T5 - NRC (1994) – 1 to 42 days; T6 - Commercial Manual – 1 to 10 and 11 to 42 days of age. *According to NRC recommendations expressed as total amino acids.

**Table 11:** Overall productive performance and carcass characteristics of European quails subjected to different nutritional plans.

Variables	T1	T2	T3	T4	T5	T6	*P-value*	CV
FI	17.3b	17.0b	20.0a	20.0a	20.1a	19.7a	<0.01	3.53
WG	6.7a	6.3b	6.0c	6.0c	5.9c	5.9c	<0.01	4.18
FCR	2.6b	2.7b	3.6a	3.6a	3.8a	3.5a	<0.01	7.92
LW	249.9ab	232.1b	267.0a	265.5a	259.1a	264.5a	<0.01	5.37
CY	75.2a	73.6ab	70.9c	70.9c	71.8bc	71.2c	<0.01	2.00
CW	188.05ab	178.87b	201.76a	200.68a	198.43a	200.68a	<0.01	6.05

T1 - Rostagno et al. (2024) – 1 to 14 and 15 to 35 days; T2 - Rostagno et al. (2024) – 1 to 35 days; T3 - Silva and Costa (2009) – 1 to 21 and 22 to 42 days; T4 - Silva and Costa (2009) – 1 to 42 days; T5 - NRC (1994) – 1 to 42 days; T6 - Commercial Manual – 1 to 10 and 11 to 42 days of age. FI = feed intake (g/quail/day); WG = weight gain (g/quail/day); FCR = feed conversion ratio (g/g); LW = live weight (g/quail); CY = carcass yield (%); CW = carcass weight (g/quail); P-value = probability value; CV = coefficient of variation (%).

Treatments T1 – Rostagno et al. (2024), T3 – Silva and Costa (2009), and T6 – Commercial Manual were formulated using multi-phase feeding programs, with two diets throughout the production cycle, allowing more precise adjustments of protein, energy, and amino acid levels according to the physiological requirements of the birds at each growth stage. In contrast, T2 – Rostagno et al. (2024), T4 – Silva and Costa (2009), and T5 – NRC (1994) used a single formulation during the entire experimental period, maintaining constant nutritional ratios throughout rearing.

Treatments formulated according to Rostagno et al. (2024), especially T1, showed lower energy:protein ratios (49.64) in the first phase and higher ratios in the second phase (56.61), associated with an adequate balance of digestible amino acids. The use of multi-phase feeding in this program likely contributed to a better alignment between nutrient supply and the birds’ requirements throughout growth.

These treatments were associated with lower feed intake (17.3 and 17.0 g/bird/day) (P<0.01) and improved feed conversion ratios (2.6 and 2.7) (P<0.01), indicating greater efficiency of nutrient utilization. Additionally, T1 – Rostagno et al. (2024) resulted in the highest carcass yield (75.2%) (P<0.01), demonstrating more efficient nutrient partitioning toward muscle tissue deposition.

Similar results were reported by Dozier et al. (2008) and Kidd and Tillman (2016), who demonstrated that diets formulated with an adequate balance of digestible amino acids improve nutrient utilization efficiency, enhance muscle tissue deposition, and increase carcass yield in fast-growing poultry.

Treatments formulated according to Silva and Costa (2009) showed distinct responses between the multi-phase program (T3) and the single-diet program (T4). Although both promoted higher body weights (267.0 and 265.5 g) (P<0.01) and greater carcass weights (P<0.01) at slaughter (201.76 and 200.68 g), feed conversion ratios remained elevated (3.6) (P<0.01). This suggests that increased body growth was not accompanied by the same efficiency of nutrient utilization observed in treatments based on Rostagno et al. (2024). Furthermore, the multi-phase program in T3 – Silva and Costa (2009) may have contributed to better nutritional adjustment across growth phases compared with the continuous use of a single diet in T4 – Silva and Costa (2009).

The treatment based on NRC (1994), formulated with a single diet throughout the experimental period, showed the highest energy:protein ratio (58), associated with lower ratios of sulfur amino acids to lysine. This treatment resulted in the poorest feed conversion ratio (3.8) (P<0.01), along with lower daily weight gain (P<0.01) and intermediate carcass yield (P<0.01). These findings indicate that diets with higher energy density relative to protein content may limit the proportional intake of essential amino acids, impairing muscle growth efficiency and favoring greater fat deposition (Dozier et al., 2008; Kidd and Tillman, 2016).

Similarly, the nutritional program based on the Commercial Manual (T6), although also employing multi-phase feeding, presented less balanced amino acid ratios, particularly for methionine + cystine and threonine relative to lysine. This was reflected in intermediate performance for body weight, feed conversion, and carcass yield. These results demonstrate that the use of multi-phase feeding programs alone does not guarantee improved productive efficiency, highlighting the importance of proper balancing among energy, protein, and digestible amino acids at each stage of production.

## Conclusion

Overall, nutritional programs markedly affected the productive performance and egg quality of Japanese quail and the growth performance and carcass yield of European quail. The Rostagno et al. (2024) program resulted in lower feed intake and improved feed conversion during Japanese quail growth and, during the laying phase, produced the highest egg production, egg mass, specific gravity, and eggshell percentage. In European quail, the multi-phase Rostagno et al. (2024) program improved feed efficiency and carcass yield, although some older programs resulted in greater body weight. These findings support the use of updated, phase-specific nutritional programs that account for genetic potential and production stage to improve nutrient utilization and productive efficiency. Further research using factorial approaches is warranted to clarify the individual and interactive effects of feeding phase, protein and energy density, and digestible amino acid balance across quail genotypes and production stages.

## Author Contribution

**Adiel Vieira de Lima** led the experimental study and was responsible for its execution, including animal management, preparation and administration of the experimental diets, collection and organization of experimental data, statistical analysis, interpretation of the results, and preparation of the original manuscript. **Danilo Vargas Gonçalves Vieira** contributed to the execution of the experiment, animal management, collection of experimental data, interpretation of the results, statistical analysis, and preparation and revision of the manuscript. **Ricardo Romão Guerra** contributed to the development and application of the intestinal histological procedures, processing and analysis of histological samples, interpretation of histological findings, and preparation of the manuscript. **Raiane dos Santos Silva** contributed to the execution of the experiment, including animal management, collection of experimental data and samples, organization and processing of the data, and literature investigation. **Carlos Henrique do Nascimento** contributed to the execution of the experiment, including animal management, collection of experimental data and samples, organization and processing of the data, and literature investigation. **Paloma Eduarda Lopes de Souza** contributed to the execution of the experiment, including animal management, collection of experimental data and samples, organization and processing of the data, and literature investigation. **Aline Beatriz Rodrigues** contributed to the execution of the experiment, including animal management, collection of experimental data and samples, organization and processing of the data, and literature investigation. **Raul da Cunha Lima Neto** contributed to the execution of the experiment, collection and processing of experimental samples, and analysis and interpretation of the intestinal histological data. **Matheus Ramalho de Lima** contributed to the execution of the experiment, collection and organization of experimental data, literature investigation, interpretation of the results, and preparation of the manuscript. **Apolônio Gomes Ribeiro** contributed to the execution of the experiment and field activities, collection and analysis of experimental data, interpretation of the results, organization of the study for publication, and preparation and revision of the manuscript. **Humberto de Araújo Brito Filho** contributed to the execution of the experiment, collection and processing of experimental samples, and analysis and interpretation of the intestinal histological data. **Vânia Maria Bernardo de Araújo** contributed to the execution of the experiment, including animal management, collection of experimental data and samples, organization and processing of the data, and literature investigation. **José de Arimatéia de Freitas Pinto** contributed to the execution of the experiment, including animal management, collection of experimental data and samples, organization and processing of the data, and literature investigation. **Lucas Rannier Ribeiro Antonino Carvalho** contributed to the execution of the experiment, research and literature investigation, development and refinement of experimental methodologies, collection and processing of data, interpretation of the results, and preparation and revision of the manuscript. **Fernando Guilherme Perazzo Costa** supervised and coordinated the study, contributed to the experimental design and methodological development, participated in the execution and interpretation of the research, and contributed to the preparation and revision of the manuscript.

## Disclosures

The authors declare that they have no other conflicts of interest.
